# Upregulation of Nrf2 Signalling and the Inhibition of Erastin-Induced Ferroptosis by Ferulic Acid in MIN6 Cells

**DOI:** 10.3390/ijms232415886

**Published:** 2022-12-14

**Authors:** Tugba Kose, Paul A. Sharp, Gladys O. Latunde-Dada

**Affiliations:** Department of Nutritional Sciences, School of Life Course and Population Sciences, King’s College London, London SE1 9NH, UK

**Keywords:** ferulic acid, ferroptosis, iron, erastin, of nuclear factor erythroid-2-related factor 2

## Abstract

Ferroptosis is a regulated cell death process characterised by the iron-dependent accumulation of oxidised polyunsaturated fatty acid-containing phospholipids. Its initiation is complicated and involves reactive oxygen species (ROS) and a loss of the activity of the lipid repair enzyme glutathione peroxidase 4 (GPX4). These play critical roles in the development of ferroptotic cell damage by lipid peroxidation. Antioxidant therapy is a promising therapeutic strategy to prevent or even reverse the progression of ferroptosis. This study was designed to demonstrate the protective effect of ferulic acid (FA) against oxidative stress and erastin-mediated ferroptosis in murine MIN6 cells. Cells were treated with FA or its metabolite ferulic acid 4-O-sulfate disodium salt (FAS) and 20 μM of erastin. Cell viability was determined by 3-(4,5-dimethyl-2-thiazolyl)-2,5-diphenyltetrazolium bromide (MTT) assay, iron levels were measured by inductively coupled plasma mass spectrometry (ICP-MS), ROS levels were determined by a dihydrodichlorofluorescein (H2DCF) cell-permeant probe, and glutathione and lipid peroxidation were assayed with commercially available kits. The phenolic acids enhanced cell viability in erastin-treated MIN6 cells in a dose-dependent manner. Furthermore, MIN6 cells exposed to erastin alone showed elevated levels of iron and ROS, glutathione (GSH) depletion, and lipid peroxidation (*p* < 0.05) compared to cells that were protected by co-treatment with FA or FAS. The treatment of MIN6 cells with FA or FAS following exposure to erastin increased the nuclear translocation of nuclear factor erythroid-2-related factor 2 (Nrf2) protein levels. Consequently, levels of its downstream antioxidant proteins, HO-1, NQO1, GCLC, and GPX4, increased. FA and FAS greatly decreased erastin-induced ferroptosis in the presence of the Nrf2 inhibitor, ML385, through the regulation of Nrf2 response genes. In conclusion, these results show that FA and FAS protect MIN6 cells from erastin-induced ferroptosis by the Nrf2 antioxidant protective mechanism.

## 1. Introduction

Iron is vital for several biological processes; however, excess iron results in oxidative stress, subsequent mitochondrial impairment, DNA damage, and lipid peroxidation, leading in particular to the dysfunction of highly metabolically active cells such as hepatocytes, cardiomyocytes, and pancreatic β cells [1].

A growing body of evidence suggests that iron-induced pancreatic β-cell impairment is involved in T2D and/or its complications [2,3], including insulin synthesis and β-cell signalling pathways [4]. Pancreatic β cells have highly active mitochondria that generate activated ROS, which, in turn, can lead to synergistic toxicity with intracellular iron. This is especially accentuated in β cells due to their intrinsically weak antioxidant defences [5,6].

Ferroptosis is a form of regulated cell death that is characterised by the iron-mediated accumulation of lipid peroxides. Erastin is a ferroptosis inducer that inhibits the glutamate–cystine antiport system Xc-. The exchange of extracellular cysteine for intracellular glutamate by system Xc- is vital for glutathione biosynthesis [7]. Glutathione neutralises reactive oxygen species (ROS) and protects cells from oxidative stress. A lack of glutathione can inhibit GPX4 activity and induce lipid peroxidation, thus leading to the disruption of cell structure, which results in cell death [8].

Ferroptosis plays a crucial role in pancreatic β-cell death due to excess iron toxicity and low antioxidant reserves [9]. Importantly, cells have numerous endogenous antioxidant defence mechanisms such as the basic leucine zipper (bZIP) transcription factor and nuclear factor erythroid-2-related factor 2 (Nrf2), which attenuates ROS and oxidative stress in cells. Nrf2 is a vital regulator of the cellular antioxidant response, controlling the expression of genes such as HO-1, NQO1, GCLC, and GPX4, which counteract oxidative and electrophilic stresses [10]. As such, Nrf2 functionality is critical for cell survival under increased metabolic and oxidative stress. Due to its multifaceted role in promoting cell survival, it is not surprising that the inhibition of Nrf2 and/or its downstream target genes are associated with a decreased responsiveness to cellular stressors and increased cell death, such as ferroptosis [11].

Ferulic acid (FA) is a plant-based phenolic acid that is commonly found in fruits, vegetables, and some beverages including coffee and beer [12]. FA has been reported to exhibit antioxidant, anti-inflammatory, anticancer, and hepatoprotective effects [13]. Its antioxidant property is due to the phenolic hydroxyl group in its structure that donates electrons to quench free radicals [14]. Furthermore, FA functions as an Nrf2 agonist and can promote the nuclear translocation and biological effects of Nrf2 [15]. Moreover, FA exhibits low toxicity in cells and higher bioavailability compared to other phenolic acids [13,14]. These properties, as well as the bioactivity potentials of FA, could be deployed as natural cell-death attenuating agents. This study, therefore, aimed to investigate the protective functions of FA and its metabolite, ferulic acid 4-O-sulfate disodium salt (FAS), against ferroptosis in MIN6 cells. The molecular antioxidant mechanism was associated with the activation of the Nrf2 pathway. These results suggest that FA and FAS may be considered therapeutic agents for the treatment and prevention of ferroptosis in MIN6 pancreatic β cells.

## 2. Results

### 2.1. FA and FAS Alleviate the Decreased Viability of MIN6 Cells Induced by Erastin

**Figure 1:**
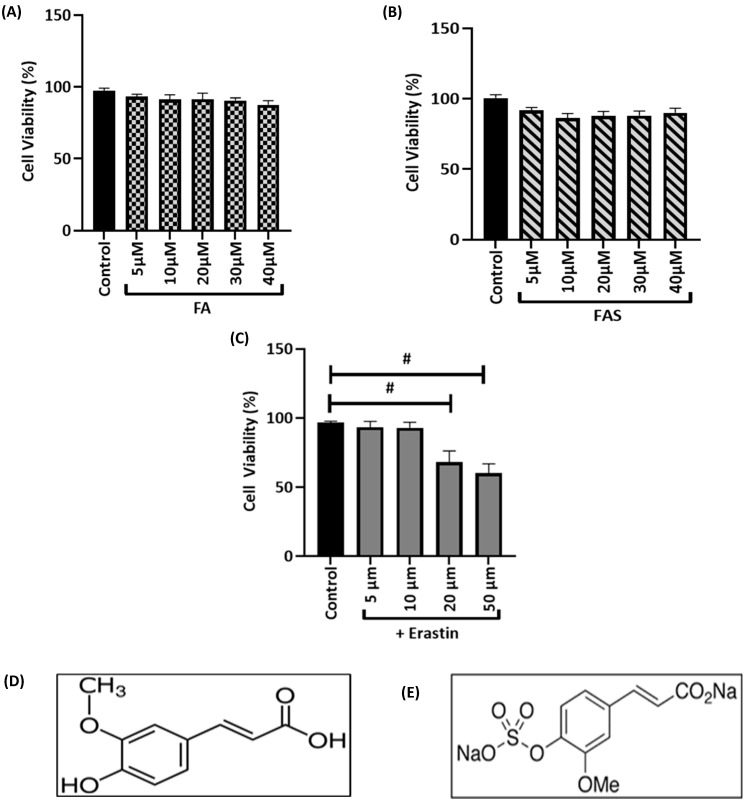
Relative cell viability of MIN6 cells against different concentrations of FA, FAS, or erastin. (**A**) MIN6 cells were exposed the different concentrations of ferulic acid (FA) for 24 h to determine its toxic concentration. (**B**) MIN6 cells were exposed to the different concentrations of ferulic acid 4-O-sulfate disodium salt (FAS) for 24 h to determine its toxic concentration. (**C**) MIN6 cells were treated with the indicated concentrations (5, 10, 20, and 50 μM) of ferroptosis inducer, erastin, for 24. Cell viability was measured by MTT assay. Data are presented as the percentage of cell viability with the error bars representing the SEM. (**D**) Chemical structure of ferulic acid (FA). (**E**) Chemical structure of ferulic acid 4-O-sulfate disodium salt (FAS). All the values are expressed as the mean ± SEM, *n* = 8. ^#^ *p* < 0.05 control vs. treatment groups. One-way ANOVA, Tukey’s post hoc test.

The protective effects of FA and FAS on MIN6 cells were investigated by MTT assay, the results indicated that treatments with FA or FAS alone (5–40 μM) did not cause any obvious cytotoxicity for 24 h (Figure 1A,B). Next, MIN6 cells were exposed to a series of erastin concentrations for 24 h. Figure 1C shows that erastin exposure reduced cell viability in a dose-dependent manner. An exposure of MIN6 cells to 20 μM of erastin for 24 h resulted in significant (*p* < 0.05) cell death compared to the untreated control group. Therefore, 24 h of treatment with 20 μM of erastin was used to induce ferroptotic injury to MIN6 cells in this study.

**Figure 2:**
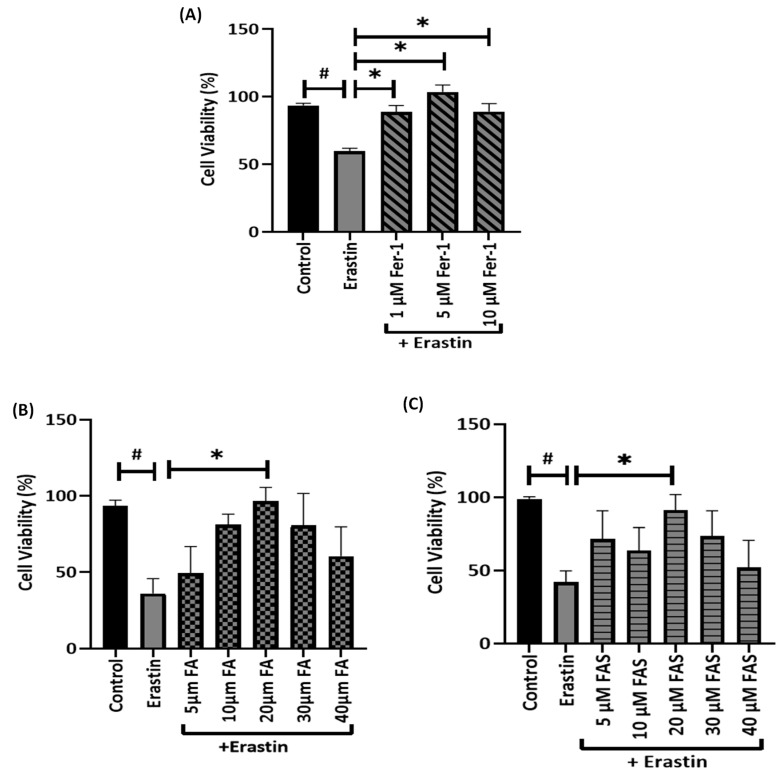
FA and FAS protect MIN6 cell viability from erastin-induced ferroptosis. MIN6 cells were exposed to ferroptosis inducer, 20 μM of erastin, in the absence or presence of (1, 5, and 10 μM) ferrostatin (Fer-1) (**A**), (5, 10, 20, 30, and 40 μM) ferulic acid (FA) (**B**), or (5, 10, 20, 30, and 40 μM) ferulic acid 4-O-sulfate disodium salt (FAS) (**C**) in a dose-dependent manner for 24 h. Cell viability was assayed to show the protective effects of FA, FAS, or Fer-1 against erastin treatment. All the values are expressed as the mean ± SEM, *n* = 8. ^#^ *p* < 0.05 control vs. treatment groups; * *p* < 0.05 erastin vs. treatment groups. One-way ANOVA, Tukey’s post hoc test.

To examine the protective effect of FA and FAS treatments on erastin-induced ferroptosis in MIN6 cells, cells were treated with or without FA and FAS (5, 10, 20, 30, and 40 μM) and 20 μM of erastin for 24 h. The concentrations of phenolic acids were determined based on research related to the antioxidant effects of FA [12]. The decrease in cell viability following exposure to erastin was abolished by 20 μM of FA or FAS treatment (Figure 2B,C; *p* < 0.05). Moreover, when MIN6 cells were treated with different concentrations of ferroptosis inhibitor Fer-1 in the presence of 20 μM of erastin, Fer-1 was able to block the cell death caused by 20 μM of erastin (Figure 2A).

### 2.2. FA and FAS Suppress Iron Accumulation and Can Act as Iron Chelators

**Figure 3:**
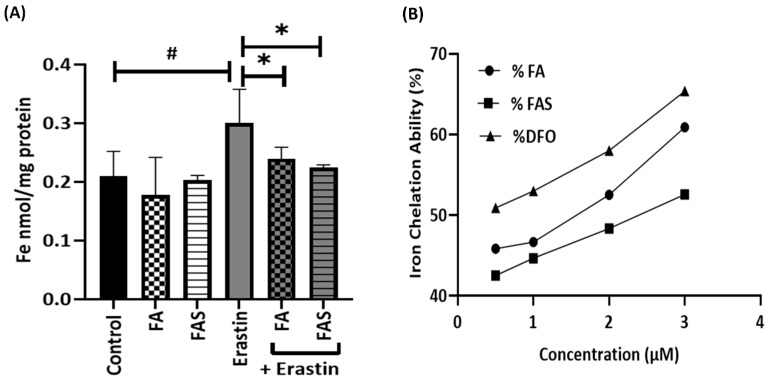
The iron-chelating capability of FA and FAS in MIN6 cells. (**A**) Cells were treated with 20 μM of erastin in the absence and presence of 20 μM of ferulic acid (FA) or ferulic acid 4-O-sulfate disodium salt (FAS) for 24 h. Then, iron concentrations were measured by ICP-MS analysis. (**B**) Different concentrations of FA, FAS, and deferoxamine (DFO) (0.5 μM, 1 μM, 2 μM, and 3 μM) were analysed to show iron chelation ability. All the values are expressed as the mean ± SEM. ^#^ *p* < 0.05 control vs. treatment groups; * *p* < 0.05 erastin only vs. treatment groups. One-way ANOVA, Tukey’s post hoc test.

Next, the role of iron in ferroptosis was studied. ICP-MS analysis showed that the concentration of iron in MIN6 cells increased after 20 μM erastin treatment by 0.3 nmol/mg cell protein which was inhibited by the co-treatment of 20 μM FA and FAS and 20 μM erastin (Figure 3A). To validate the effect of FA and FAS on cellular iron levels, we measured their capacity to act as iron chelators. Since cell death caused by ferroptosis is highly related to free iron in vivo, iron chelation is inhibitory to ferroptosis [16]. Phenolic acids might function similarly. As shown in Figure 3B, FAS resulted in 46% iron chelation ability at a 1 μM Fe concentration and 50% at a 3 μM Fe concentration, while that of FA was 42% and 49%. This showed that both phenolic acids have similar iron chelation abilities. Moreover, they indicated an upward trend of chelating potential with increasing doses of iron compared with the iron chelator DFO (Figure 3B).

### 2.3. Treatments of FA and FAS Limit Lipid Peroxidation and ROS Production by Increasing GSH Antioxidant Levels

**Figure 4:**
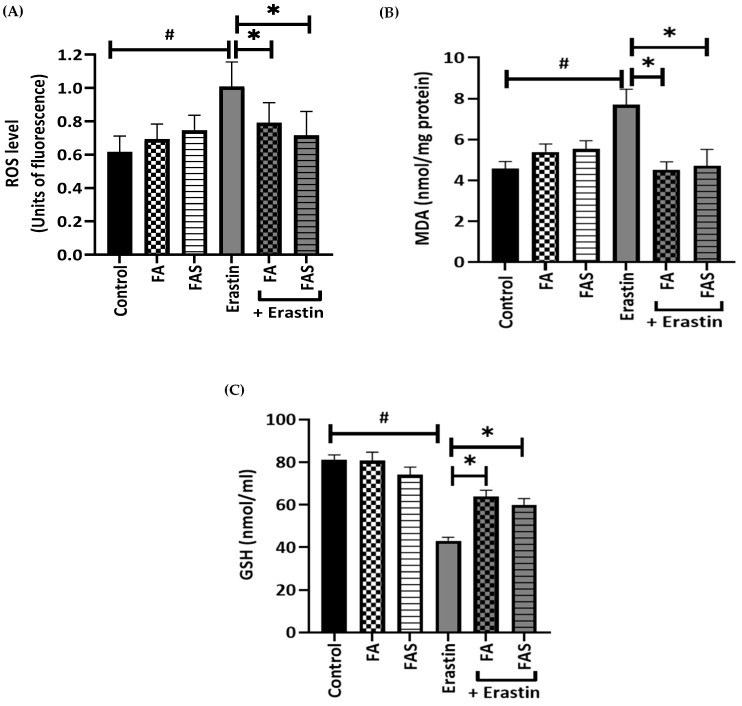
FA and FAS limit lipid peroxidation and ROS production by increasing GSH levels. Cells were treated with 20 μM of erastin in the absence and presence of 20 μM of ferulic acid (FA) or ferulic acid 4-O-sulfate disodium salt (FAS) for 24 h. Then, cellular reactive oxygen species (ROS) were estimated with a dihydrodichlorofluorescein (H2DCF) cell-permeant probe (**A**), lipid peroxidation levels were measured by malondialdehyde (MDA) assay (**B**), and glutathione (GSH) amounts were measured by total GSH assay (**C**). All the values are expressed as the mean ± SEM. ^#^ *p* < 0.05 control vs. treatment groups; * *p* < 0.05 erastin only vs. treatment groups. One-way ANOVA, Tukey’s post hoc test.

To examine whether FA and FAS might protect MIN6 cells from ferroptosis, pancreatic MIN6 cells were treated with 20 μM of erastin. Intracellular ROS levels were detected in erastin-treated MIN6 cells by measuring 2′,7′ –dichlorofluorescein (DCF) by flow cytometry. Compared with the control group, the intracellular ROS levels of MIN6 cells were significantly increased following treatment with erastin (*p* < 0.05). Notably, co-treatment with FA and FAS effectively reduced erastin-induced ROS accumulation, although treatment with FA and FAS in the absence of erastin did not change intracellular ROS levels in MIN6 cells (Figure 4A).

MDA is an indicator of membrane lipid peroxidation, which indirectly reflects the degree of damage to a cell [17]. In order to validate the effects of FA and FAS on the antioxidant capacity of erastin-induced MIN6 cell injury, MDA levels were measured. As shown in Figure 4B, when compared with the erastin-alone treatment group, the MDA levels in FA or FAS and erastin co-treatment groups were significantly decreased. During ferroptosis, Fe^2+^ converts membrane lipids into hydroperoxides, and GPX4 converts lipid hydroperoxides into the corresponding alcohol using GSH as a cofactor. GSH maintains catalytic and regulatory thiol groups that play a role in maintaining the intracellular redox balance by eliminating free radicals and thereby reducing oxidative damage [18]. As shown in Figure 4C, the erastin-alone treatment group demonstrated significantly decreased GSH levels, whereas FA and FAS treatment in MIN6 cells exposed to erastin increased GSH levels. FA and FAS treatments can trigger antioxidant pathways, thus increasing antioxidant GSH levels in MIN6 cells. The balance between GSH and ROS is essential for maintaining normal cellular functions. Therefore, it could be concluded that FA and FAS downregulate the levels of ROS by promoting GSH and inhibiting iron and MDA levels, thereby alleviating ferroptosis.

### 2.4. Phenolic Acids Activate Nrf2 Pathway in Erastin-Induced MIN6 Cells

**Figure 5:**
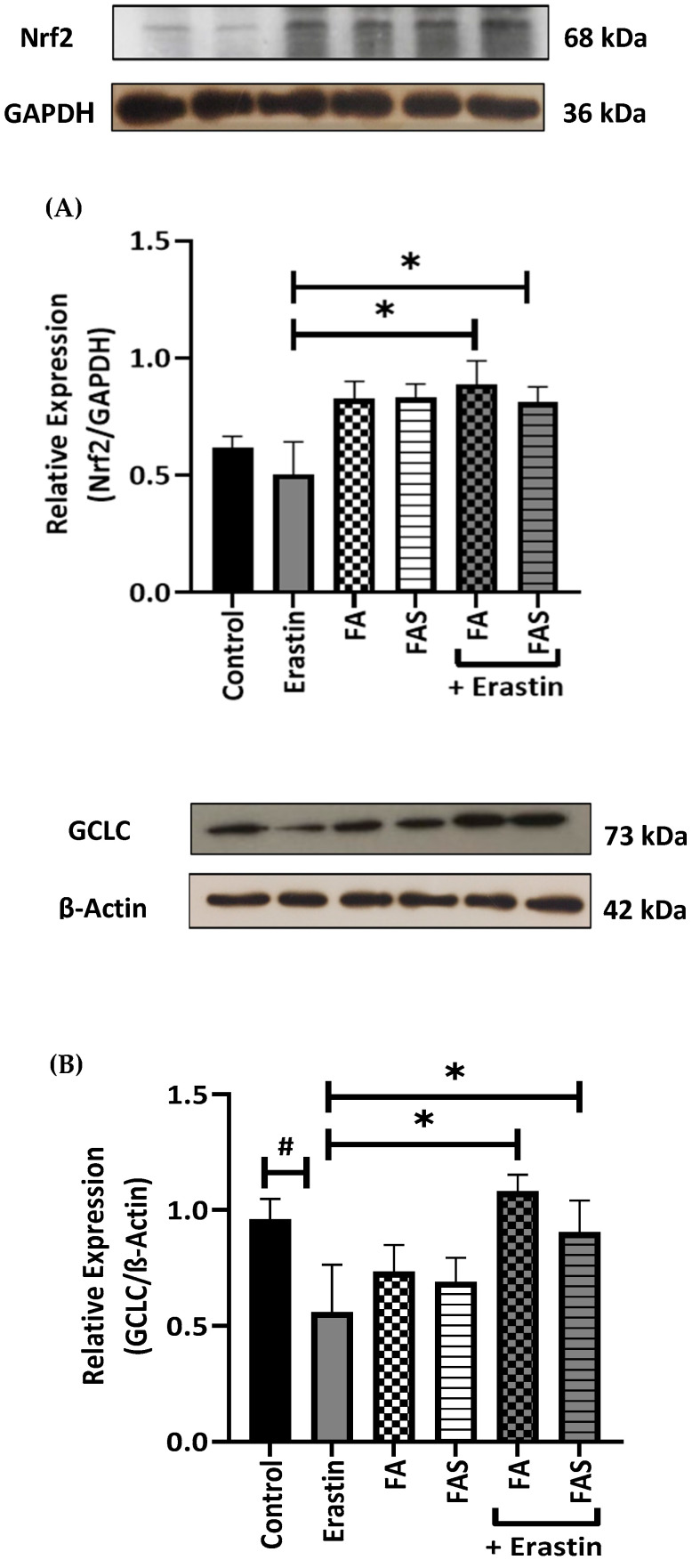

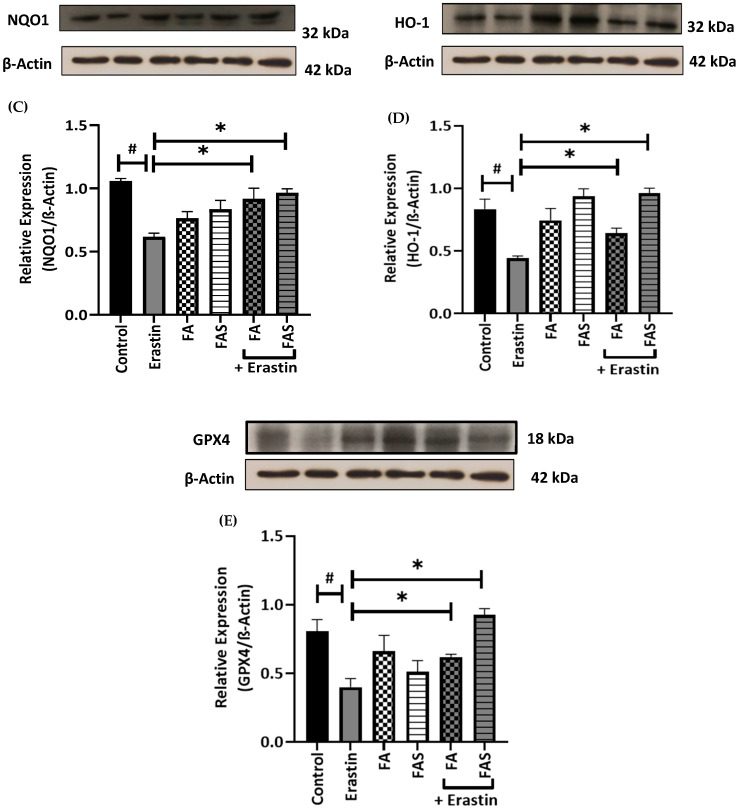
FA and FAS activate the Nrf2 pathway. Cells were treated with 20 μM of erastin in the absence and presence of 20 μM of ferulic acid (FA) or ferulic acid 4-O-sulfate disodium salt (FAS) for 24 h. Total cell lysates were prepared and subjected to Western blot analysis to monitor the expression levels of Nrf2 (**A**) and its downstream antioxidant proteins, including GCLC (**B**), NQO1 (**C**), HO-1 (**D**) and GPX4 (**E**). GAPDH and β-Actin served as internal controls. The quantitative results of the protein expressions in pancreatic β cells were determined by greyscale analysis using the ImageJ software. All the values are expressed as the mean ± SEM. ^#^ *p* < 0.05 control vs. treatment groups; * *p* < 0.05 erastin only vs. treatment groups. One-way ANOVA, Tukey’s post hoc test.

To further analyse the cytoprotective role of FA and FAS in erastin-induced ferroptosis in MIN6 cells, the protein levels of Nrf2, GPX4, HO-1, GCLC, and NQO1 were determined. Cells treated with erastin alone showed significantly reduced protein expression levels of Nrf2, GPX4, HO-1, GCLC, and NQO1 (*p* < 0.05) (Figure 5). On the contrary, co-treatment with FA and FAS significantly increased the expression of Nrf2, GPX4, HO-1, GCLC, and NQO1 in erastin-induced cells, thus suggesting the activation of the Nrf2 signalling pathway by FA and FAS.

### 2.5. Nrf2 Activation Shows the Protection against Ferroptosis in ML385-Treated MIN6 Cells

**Figure 6:**
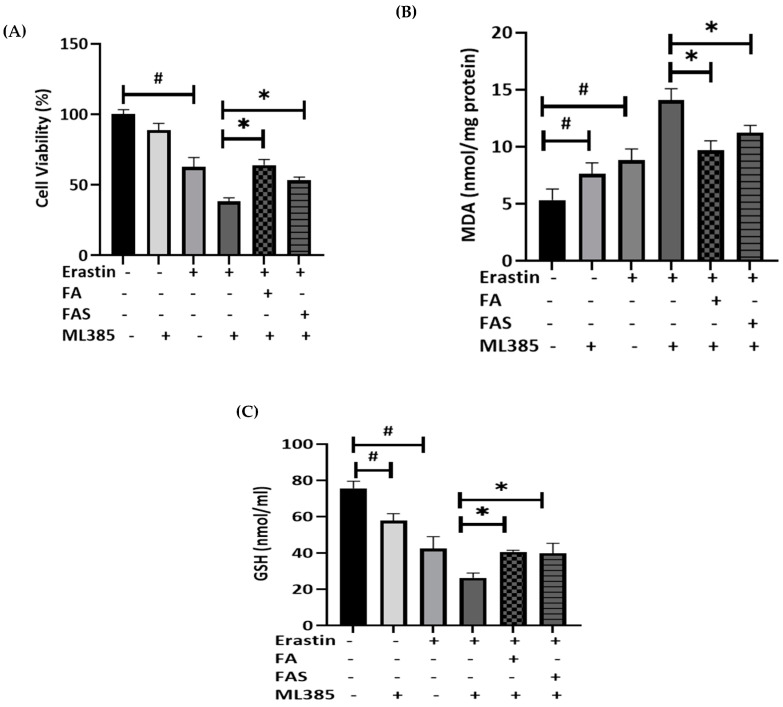
Phenolic acids protect MIN6 cells treated with ML385, an Nrf2 inhibitor against ferroptosis markers. Cells were treated with 10 μM of Nrf2 inhibitor, ML385, for 2 h before ferroptosis treatment. Then, MIN6 cells were exposed to 20 μM of erastin in the absence and presence of 20 μM of ferulic acid (FA) or ferulic acid 4-O-sulfate disodium salt (FAS) for 24 h. Cell viability was presented by MTT assay (**A**), lipid peroxidation was analysed by MDA assay (**B**), and total glutathione (GSH) amounts (**C**) was measured by GSH assay. All the values are expressed with the mean ± SEM. ^#^ *p* < 0.05 controls vs. treatment groups; * *p* < 0.05 erastin only vs. treatment groups. One-way ANOVA, Tukey’s post hoc test.

To further confirm the protective effect of Nrf2 against ferroptosis, MIN6 cells were treated with ML385, an Nrf2 inhibitor. The results revealed that ML385, when combined with erastin treatment, significantly reduced cell viability by 38% compared with the control group (*p* < 0.05) (Figure 6A). Furthermore, FA and FAS attenuated the toxic effect of erastin in ML385-pretreated MIN6 cells. In terms of the antioxidant roles of FA and FAS, both phenolic acids showed significant repressive effects on lipid peroxidation levels in erastin-induced MIN6 cells in the presence of ML385 pre-treatment (Figure 6B) (*p* < 0.05). Decreased GSH levels by the erastin and ML385 treatment of the cells were attenuated by the phenolic acids (Figure 6C) (*p* < 0.05).

**Figure 7:**
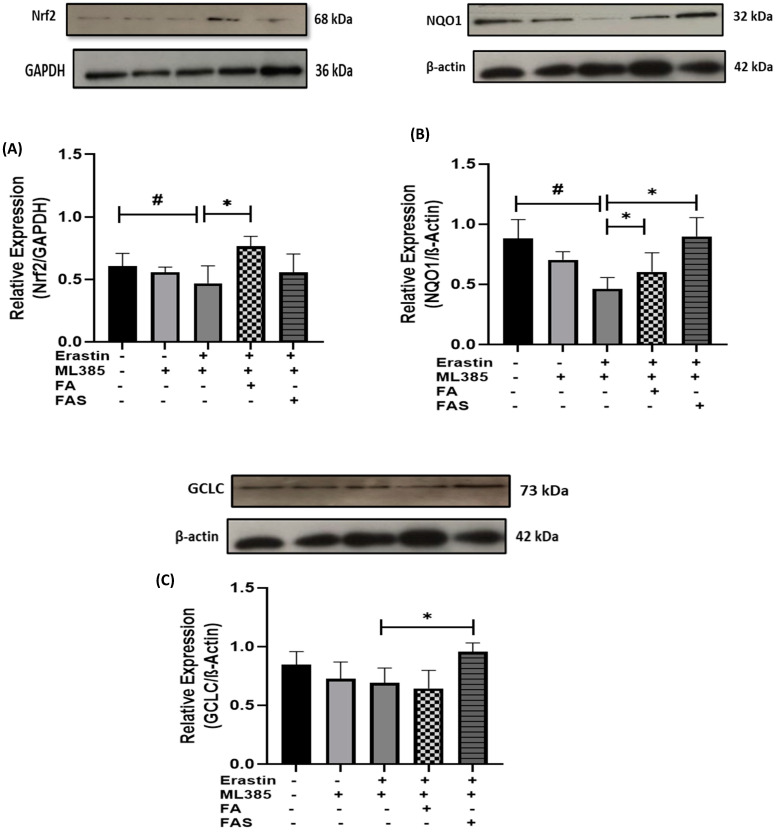
Nrf2 activation confers protection against ferroptosis in ML385-treated MIN6 cells. Cells were treated with 10 μM of Nrf2 inhibitor, ML385, for 2 h before ferroptosis treatment. Then, MIN6 cells were exposed to 20 μM of erastin in the absence and presence of 20 μM of ferulic acid (FA) or ferulic acid 4-O-sulfate disodium salt (FAS) for 24 h. The expressions of Nrf2 (**A**), NQO1 (**B**), and GCLC (**C**) were measured by Western blotting. GAPDH and β-Actin served as internal controls. The quantitative results of the protein expressions in pancreatic β cells were determined by greyscale analysis using the ImageJ software. All the values are expressed as the mean ± SEM. ^#^ *p* < 0.05 control vs. treatment groups; * *p* < 0.05 erastin only vs. treatment groups. One-way ANOVA, Tukey’s post hoc test. All the values are expressed as the mean ± SEM. ^#^ *p* < 0.05 control vs. treatment groups; * *p* < 0.05 erastin only vs. treatment groups. One-way ANOVA, Tukey’s post hoc test.

After the pre-treatment of MIN6 cells with ML385, the protein levels of Nrf2 and its downstream genes, including GCLC and NQO1, were increased in the erastin, FA, or FAS co-treatment groups compared with erastin alone (*p* < 0.05) (Figure 7). Collectively, these data indicate that the cytoprotective effects of FA and FAS against ferroptosis can be achieved by activating Nrf2.

### 2.6. Phenolic Acids Increased Insulin Secretion in Erastin-Induced Ferroptotic MIN6 Cells

**Figure 8:**
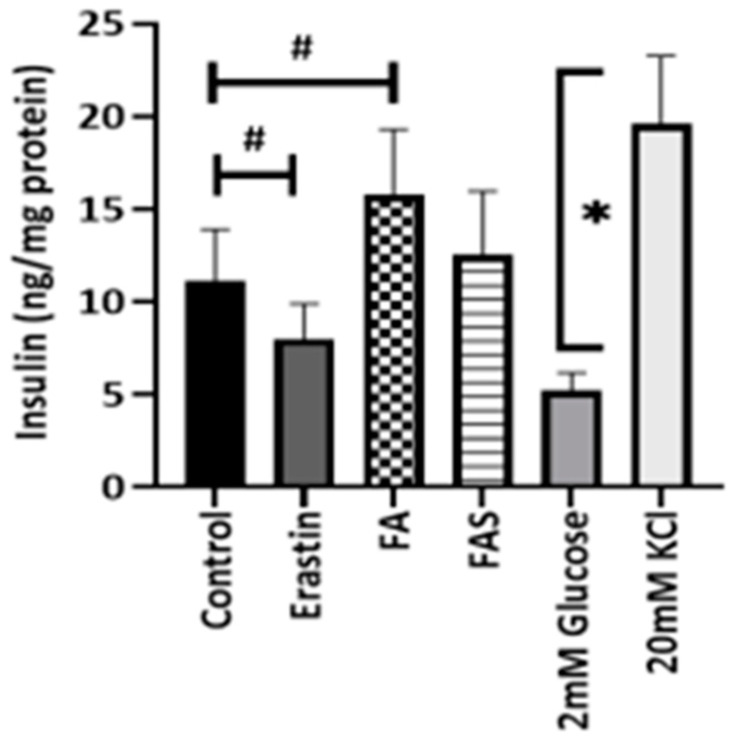
Phenolic acids and ferroptosis inducer affect insulin secretion in MIN6 β cells. Mouse MIN6 β cells were treated with 20 μM of ferulic acid (FA), ferulic acid 4-O-sulfate disodium salt (FAS), or ferroptosis inducer, erastin, for 3 h under 20 mmol/L glucose conditions. Supernatants from triplicate samples were analysed for responses to insulin secretion in MIN6 cells. All the values are expressed as the mean ± SEM. ^#^ *p* < 0.05 controls vs. treatment groups; * *p* < 0.05 2 mM glucose concentration vs. 20 mM glucose concentration groups. One-way ANOVA, Tukey’s post hoc test.

In keeping with its cytotoxic effects, the ferroptosis inducer, erastin, significantly decreased insulin secretion (*p* < 0.05) in MIN6 cells. However, FA effected approximately a 1.5-fold significant increase in insulin levels in MIN6 cells (*p* < 0.05). The effect of FAS, however, was only modest on the enhancement of insulin secretion (Figure 8) (*p* < 0.05). Under similar experimental conditions, the depolarising agent KCl (20 mmol/L) as a positive control induced a 4.2-fold increase in insulin secretion compared with the low-glucose-level group (*p* < 0.05).

## 3. Discussion

Novel therapeutic measures are employing natural products to counteract the side effects of synthetic medications used in the treatment of cancer, metabolic disorders, and T2D. Polyphenols and phenolic acids provide a new perspective for developing novel approaches against ferroptosis-induced cell damage. The induction of antioxidant pathways such as Nrf2 by phenolic acids is considered a promising therapy to prevent ferroptosis. Therefore, this study investigated the underlying mechanisms of how FA might exert cytoprotective effects against ferroptosis via stimulating the Nrf2 pathway.

Pancreatic MIN6 cells treated with erastin is a simple and feasible in vitro cell model to study ferroptosis, which can be effectively inhibited by natural antioxidants. Moreover, MIN6 cells are a proven model for glucose-stimulated insulin secretion (GSIS) activity and vulnerability to ROS-related oxidative stress that typify the islets of Langerhans [19,20,21]. Previous reports have shown that FA is an effective scavenger of free radicals such as hydroxyl radical (OH), peroxyl radical (RO2), and hypochlorous acid (HOCl), and it has also been approved in certain countries as a food additive to prevent lipid peroxidation [22]. In this study, FA prevented elastin-induced ferroptotic cell death in MIN6 cells to almost the same level as the ferroptosis inhibitor Fer-1 (Figure 2), suggesting that FA can exert strong antioxidant activity to neutralise the effect of ferroptosis inducers.

Ferroptosis results from iron-dependent lipid peroxide accumulation; thus, iron chelation therapy is a protective approach to prevent ferroptotic activity in cells. Indeed, labile-free iron causes oxidative stress and the promotion of lipid peroxide accumulation. Hence, iron chelation to avert ROS generation could be employed to prevent lipid peroxide accumulation and ferroptosis [23]. This study showed that both FA and FAS had an increasing iron-chelating ability, which was dose-dependent. That was in accordance with the study reported by Ferlazzo et al. [24]. In their study, two juice extracts from bergamot and orange, which are abundant in polyphenols, showed iron-chelating properties in epithelial A549 cells treated with 200 and/or 400 μM of Fe^3+^ [24]. Furthermore, Dixon et al. showed that co-treatment with the iron chelator deferoxamine (DFO) abrogated erastin-induced cell death in fibrosarcoma HT-1080 cells [25]. Remarkably, FA was recently reported to ameliorate myocardial ischemia/reperfusion (I/R) injury-induced ferroptosis in rats by activating AMPKa2 [26]. In the current study, co-treatments with phenolic acids suppressed iron accumulation, which was mediated by erastin treatment, in MIN6 pancreatic cells (Figure 3A), corroborating previous findings that demonstrated that baicalein decreased iron levels in erastin-induced PANC1 and BxPc3 cells and decreased ferroptosis [9]. Iron accumulation in MIN6 cells by erastin treatment would generate reactive ferrous species via the Fenton reaction to induce ferroptosis and lipid peroxidation.

This study found that FA inhibited MDA levels caused by iron accumulation by promoting GPX4 expression (Figure 4). The inhibition of GPX4 initiates uncontrolled polyunsaturated fatty acid oxidation and fatty acid radical generation, thereby causing ferroptotic cell death, suggesting that ferroptosis is triggered mainly by the reduced detoxification of lipid peroxides by the enzymatic activity of GPX4 [27] or loss of this capacity [28]. This study is consistent with our previous results that curcumin and (-)- epigallocatechin-3-gallate exerted a decrease in MDA accumulation in pancreatic cells treated with erastin [29].

An increased level of GSH is an indication of Nrf2 activation; thus, GSH levels in cells may be considered a significant factor underlying the protection associated with Nrf2 activation [30]. GSH not only produces reducing equivalents, which are necessary for the conversion of H_2_O_2_ and lipid peroxides to water and lipid alcohols, but also has an essential role in the protection of protein sulfhydration against oxidation [31]. This study exhibited that erastin blocked the glutamate-induced downregulation of GPX4 by a decrease in GSH levels while FA and FAS treatments increased GSH levels and, in turn, GPX activity. Similar to this result, the level of GSH dropped dramatically with the addition of oxidative stress inducer tert-butyl hydroperoxide (t-BOOH), and the decrease in GSH was suppressed significantly after exposure to epicatechin [32]. Moreover, consistent with our findings, FA treatment led to a reduction in MDA and iron levels and an increase in GSH levels in sepsis-induced BALB/c mice [33], thus demonstrating the ameliorative effects of FA in ferroptosis-mediated alveolar epithelial barrier dysfunction by activating the Nrf2 pathway.

Nrf2 regulation serves a key role in modulating antioxidant enzymes and is also an essential part of maintaining oxidative and antioxidative homeostasis and alleviating oxidative stress damage [34]. NQO1, GCLC, GPX4, and HO-1 are some downstream genes of Nrf2 regulation, and this has a significant role in protecting cells from oxidative stress [34]. The present study proposed that the potential antioxidative mechanism of FA may comprise the activation of the Nrf2 signalling pathway in pancreatic MIN6 cells. Our results suggest that an increase in Nrf2 protein expression after FA treatment in pancreatic MIN6 cells exposed to ferroptotic activity reversed the inhibitory effects of erastin on the protein expression of NQO1, GCLC, GPX4, and HO-1. In addition, the effects of FA and FAS on MIN6 cells treated with ML385 (an inhibitor of Nrf2 activity) were investigated in the present study. The results revealed that ML385 exhibited no toxicity to MIN6 cells. At the molecular level, Nrf2 inhibitor ML385 enhanced the impact of erastin-induced cell damage via increasing MDA levels. Nrf2 inhibition with ML385 weakened the improvement effects of the phenolic acids on ferroptosis. Furthermore, FA and FAS treatment resulted in the increased protein expression of Nrf2, NQO1, GCLC, GPX4, and HO-1 in MIN6 cells treated with ML385 against erastin-induced ferroptosis. Phenolic acids exerted their inhibitory effect by blocking Nrf2–ML385 interaction, thereby limiting ferroptosis. Therefore, the activation of Nrf2 signalling may be closely associated with the protective effects of FA.

The level of insulin release in cell-culture models can be used as a biochemical marker to predict iron-related pancreatic damage and to assess the effects of natural or synthetic iron chelators [35]. This study indicated that ferroptosis is associated with decreased insulin secretion, whereas FA could increase insulin secretion in glucose-stimulated pancreatic MIN6 cells. A study by Saji et al. showed that rice bran phenolic extracts significantly increased glucose-stimulated insulin secretion in INS-1E cells [36]. However, an increased concentration of rice bran phenolic extracts decreased insulin secretion, suggesting that the determination of certain concentrations of natural products is vital for antioxidant activity. In comparison, the inhibitory effect of insulin secretion in a pancreatic β-cell line by natural products was reported [37]. High doses of iron chelators and free radical scavengers were found to be toxic to rat pancreatic cells [38]. Accordingly, depending on the concentration of natural products, they can act either as a prooxidant or potential antioxidants. Additionally, this effect might be influenced by the redox state of iron and glucose sensitivities of different pancreatic insulinoma β cells.

The physiologic importance of FA, and notably its antioxidant property, depends upon its absorption and subsequent interaction with target tissues. The bioavailability of FA has been addressed in several studies quantified as urinary excretion with variable results: from low to high bioavailability (0.4–98%), in part depending on the food source [39,40]. For instance, by the consumption of cereal products, particularly bran, FA presented a low bioavailability: 3% in humans [41], 2.5–5% in rats [42], and even lower, 0.4–0.5%, from corn bran in rats [43]. The bioavailability of FA was somewhat higher from other food matrices such as tomato, 11–25% [44], or rye bread, 28% [45], while from beer, FA was highly bioavailable, at 19–98% [39]. The concentration of FA in plasma under normal physiological conditions is in the range of 0.2–2 μM of FA [46]. To further elucidate the potential role of FA on health benefits, studies on the bioavailability of FA from its natural food matrices are needed.

In summary, the present study proposed the novel functions of FA, which protected MIN6 cells against erastin-induced ferroptotic damage by promoting cell viability. The molecular mechanism is possibly due to the activation of the Nrf2-induced expression of antioxidant proteins. The protective effect of a range of phenolic iron chelators against erastin-induced ferroptosis has also been observed in preliminary studies in human PANC1 cells, validating our findings in MIN6 cells. Future experiments are planned to confirm the protective function of FA against ferroptosis in mouse models and human trials.

## 4. Materials and Methods

### 4.1. Chemicals

The antibody to NRF2 was obtained from Santa Cruz Biotechnology (Wembley, UK) and GPX4 was purchased from R&D Systems (Abingdon, UK). The antibodies to NQO1, GCLC, HO-1 and β-Actin were obtained from Abcam (Cambridge, UK). Erastin and ferrostatin-1 (Fer-1) were purchased from Bertin Bioreagent (Montigny-le-Bretonneux, France). Deferoxamine, ferrozine and β-mercaptoethanol were purchased from Sigma Aldrich (Dorset, UK). All other reagents were procured from Sigma Aldrich (Dorset, UK) unless specified.

### 4.2. MIN6 Cell Culture

The mouse MIN6 pancreatic β-cell line was used in this study [47]. The cells (<30 passages) were routinely cultured in T25-cm^2^ plastic flasks. MIN6 cells were maintained in Dulbecco’s Modified Eagle’s Medium (DMEM) containing 15% heat-inactivated fetal bovine serum (FBS), 100 U/mL penicillin, and 0.1 mg/mL streptomycin. Cells were kept at 37 °C under a humidified atmosphere containing 5% CO_2_. The medium was changed twice a week. Cells were used for experimentation or split when 80–90% confluent. For experiments, MIN6 cells were exposed to erastin and/or phenolic acids for times indicated below. Control groups were treated with 0.01% DMSO.

### 4.3. Cell Viability Assay

The protective effects of FA and FAS against iron-induced cell death were investigated in MIN6 cells. Cellular metabolic activity was measured using the 3-(4,5-dimethyl-2-thiazolyl)-2,5-diphenyltetrazolium bromide (MTT) assay in a 96-well plate. MIN6 cells were seeded at a density of 5 × 10^4^ cells per well and cotreated with 20 μM of FA or FAS under exposure to erastin for 24 h. The control group was treated with 0.01% DMSO and this was done to normalise the effects (expressed as a percentage) of the treatment groups. Following this, 100 µL of fresh DMEM along with 10 μL of MTT solution (5 mg/mL in sterile phosphate buffer saline) were added to each well. After incubating for 3 h at 37 °C, 100 μL of a solubilisation buffer, dimethyl sulfoxide (DMSO), was added and incubated for 15 min at room temperature. To determine the MTT reaction in the cells, optical density was read in a microplate reader (Bio-Tek ELx800) at 490 nm. Cell viability was expressed as a percentage of the controls [48].

### 4.4. Cellular Iron Levels

Inductively coupled plasma mass spectrometry (ICP-MS) analysis of total cellular iron levels was performed. Cell pellets collected for metal analysis by ICP-MS were re-suspended in 200 μL of 50 mM NaOH. Concentrated 68% HNO_3_ (nitric acid) was added to the samples and the samples were then heated for 3 h at 80 °C to complete the digestion. Measurements were made using an Agilent ICPMS 7700 x series ICPMS instrument under operating conditions suitable for routine multi-element analysis.

### 4.5. Measurement of Intracellular Reactive Oxygen Species

Reactive oxygen species were monitored using a dihydrodichlorofluorescein (H2DCF) cell-permeant probe according to the manufacturer’s recommendations. Briefly, MIN6 cells from different groups were collected and washed with PBS and then incubated for 90 min in the dark at 37 °C in PBS containing 10 μmol/L of H2DCF. The level of ROS generated was measured using flow cytometry based on the fluorescence intensity of DCF at 525 nm after excitation at 485 nm. The levels of ROS were expressed as units of fluorescence compared with that of the control group.

### 4.6. Lipid Peroxidation Assay

The concentration of malondialdehyde (MDA), one of the endpoint products of lipid peroxidation, was measured using the lipid peroxidation colourimetric assay kit purchased from Cohesion Biosciences (London, UK) according to the manufacturer’s instructions. The absorbance of the supernatant was determined at 532 and 600 nm. MDA levels were normalised against protein content and expressed as nmol/mg protein.

### 4.7. GLUTATHIONE ASSAY

The GSH concentration in cell lysates was measured by using a glutathione assay kit purchased from Sigma Aldrich (Dorset, UK) according to the manufacturer’s instructions. The absorbance of the mixtures was read at 412 nm at 1 min intervals 5 times. The level of GSH was expressed as nmol/mL.

### 4.8. Iron Chelation Assay

The ferrous iron-chelating potentials or capacity of polyphenols including ferulic acid (FA) and 4-O-sulfate disodium salt (FAS), compared with DFO, were determined with a ferrozine assay [49]. Briefly, different concentrations (2.5, 5, 10, 20, 40, 80 µL) of DFO were mixed with 30 µM of FeSO_4_ and left to stand for 30 min at room temperature. Ferrozine (75 µM) was added to the mixture and left for a further 30 min at room temperature. After incubation, absorbance was measured at 562 nm. The control was FeSO_4_ plus ferrozine, and FeSO_4_ plus FA, FAS, and DFO were used as a blank. The ability of FA, FAS, or DFO to chelate iron was calculated using the equation below:

Iron chelating ability (%) = (control − (treatment-blank))/(control)

### 4.9. Insulin Secretion Assay

MIN6 β cells were seeded at a density of 4 × 10^6^ cells/well in a 12-well plate for 24 h at 37 °C in an atmosphere of 5% CO_2_. Cells were washed three times with glucose-free Krebs buffer and then incubated in 0.5% BSA Krebs buffer (2 mmol/L glucose) for 1 h at 37 °C in an atmosphere of 5% CO_2_. Cells were again washed three times with glucose-free Krebs buffer. Afterwards, cells were cultured in 0.05% BSA Krebs buffer (2 mmol/L glucose or 20 mmol/L KCl) and treated with FA, FAS, and erastin at the indicated doses for 3 h in the presence of 20 mmol/L glucose. Supernatants were collected for insulin measurements using the anti-rat/mouse insulin ELISA kit (Millipore, Burlington, MA, USA; EZRMI-13K) with mouse or rat insulin as a standard in accordance with the manufacturer’s instructions. The absorbance was read at 450 nm using a microplate reader (Bio-Tek, Winooski, VT, USA; ELx800).

### 4.10. Western Blot

MIN6 cells were lysed with RIPA Buffer (Tris/Cl (pH 7.6); 100 mmol/L, EDTA; 5 mmol/L, NaCl; 50 mmol/L, β-glycerophosphate; 50 mmol/L, NaF; 50 mmol/L, Na_3_VO_4_; 0.1 mmol/L, NP-40; sodium deoxycholate; 0.5%) and supplemented with Protease Inhibitor Cocktails (Thermo Scientific, Dartford, UK). Protein concentration was determined using Bio-Rad reagents (Bio-Rad Laboratories, Hercules, CA, USA). Twenty micrograms (20 μg) of protein extracts were loaded onto a 12% gel in SDS polyacrylamide gels (SDS-PAGE). The resolved proteins were transferred onto PVDF membranes and blocked with 5% non-fat dry milk in TBST buffer (20 mM Tris–Base, pH 7.4, 500 mM NaCl, 0.1% Tween20). The membranes were probed with primary NQO1, GCLC, GPX4, HO-1, and Nrf2 antibodies and a β-Actin secondary antibody diluted in TBST and incubated overnight at 4 °C. Afterwards, the membranes were probed with HRP-conjugated secondary antibody (R&D Systems, Abingdon, UK) for 1 h at room temperature. Cross-reactivity was observed with peroxidase-linked anti-IgG by using Clarity Western ECL Substrate (Watford, UK). The densitometry of the band intensities was determined using Image J software (National Institute of Health, Bethesda, MD, USA).

### 4.11. Statistical Analysis

Statistical analysis was performed by GraphPad Prism 9.0 (GraphPad Software, San Diego, CA, USA) using one-way analysis of variance (ANOVA) and Tukey’s multiple comparisons post hoc test to compare the means of the experimental groups. Data represent duplicate measurements from three independent experiments. All the values are expressed as the mean ± SEM. The test was considered significant when a *p*-value was less than 0.05.

## Data Availability

Not applicable.

## Abbreviations

DFO, deferoxamine; DMEM, Dulbecco’s Modified Eagle Medium; FA, ferulic acid; FAS, ferulic acid 4-O-sulfate disodium salt; Fer-1, ferrostatin-1; GCLC, glutamate–cysteine ligase catalytic subunit; GSH, glutathione peroxidase; GPX4, glutathione peroxidase 4; HO-1, heme oxygenase-1; MDA, malondialdehyde; Nrf2, nuclear factor erythroid 2-related factor 2; NQO1, NAD(P)H quinone dehydrogenase 1; ROS, reactive oxygen species; SOD, superoxide dismutase.
